# Generation and characterization of UL41 null pseudorabies virus variant in vitro and in vivo

**DOI:** 10.1186/s12985-018-1025-4

**Published:** 2018-08-02

**Authors:** Chao Ye, Jing Chen, Tao Wang, Jingjing Xu, Hao Zheng, Jiqiang Wu, Guoxin Li, Zhiqing Yu, Wu Tong, Xuefei Cheng, Shasha Zhou, Guangzhi Tong

**Affiliations:** 1 Shanghai Veterinary Research Institute, Chinese Academy of Agricultural Sciences, No. 518, Ziyue Road, Minhang District, Shanghai, 200241 People’s Republic of China; 2 Jiangsu Co-innovation Center for Prevention and Control of Important Animal Infectious Diseases and Zoonoses, Yangzhou, 225009 Jiangsu China

**Keywords:** CRISPR/Cas9, Gibson assembly, Pseudorabies virus, UL41, Vhs activity

## Abstract

**Background:**

The alphaherpesvirus virion host shutoff (vhs) gene, UL41, can induce degradation of host mRNAs and shut off host protein synthesis. The roles of vhs in HSV-1 and HSV-2 have been studied extensively in previous studies, however, relatively little is known about the vhs protein of PRV.

**Methods:**

A novel method combining CRISPR/Cas9 and Gibson assembly was developed to generate UL41 null PRV variant. The properties of UL41 null PRV in vitro and in vivo were further characterized. And the vhs activity of UL41 protein of PRV variant was evaluated by luciferase assay, Western-blot and RT-qPCR.

**Results:**

Gibson assembly based on homologous recombination can accomplish one-step insertion of viral DNA fragments into donor plasmids efficiently (> 80%). Cas9/gRNA further largely enhanced the efficiency of homologous recombination. Using this method we were able to rapidly generate the UL41 null and revertant viruses of PRV variant. Compared to wild type (JS-2012), the UL41 null virus showed significantly smaller plaques and lower titers in Vero cells and impaired lethality and neuroinvasion in mice. Further the UL41 protein from different PRV strains exhibited unequal vhs activity in vitro, which of JS-2012 showed significantly weaker vhs activity than that of European-American strains. In addition UL41 null virus can also significantly decrease the expression of host genes during the early period of infection, which suggests other viral factors may be also involved in host shutoff.

**Conclusions:**

CRISPR/Cas9 combined with Gibson assembly efficiently generated UL41 null PRV. Compared to wild type, UL41 null PRV showed impaired both replication capability in vitro and neuroinvasion in vivo. Further UL41 protein of PRV variant showed significantly weaker vhs activity than that of PRV SC (European-American-like strain), suggesting the deficiency of vhs activity by the PRV variant UL41 protein.

**Electronic supplementary material:**

The online version of this article (10.1186/s12985-018-1025-4) contains supplementary material, which is available to authorized users.

## Background

Pseudorabies virus (PRV) is a member of the *alphaherpesviridae* subfamily [1]. It is the etiologic agent of Aujeszky’s disease, which causes severe nervous system disorders in newborn piglets and reproductive failure in sows [2]. The combined DIVA (differentiating infected from vaccinated animals) vaccination strategy, has allowed for PRV to be eradicated or well controlled in many countries, mostly in Europe, the United States, and New Zealand, with occurrence of sporadic outbreaks only [3]. Recently, however, new PRV variants have emerged on Bartha-K61 vaccinated pig farms in China, displaying enhanced virulence in pigs and resulting in substantial economic losses [4, 5]. Genomic analysis of PRV variants isolated from these outbreaks has shown that they are evolutionarily divergent from European-American strains [6].

The genome of PRV consists of a linear double-stranded DNA of approximately 150 kb, which contains 70 genes whose protein products have not been well characterized yet [2]. Site-directed mutagenesis by homologous recombination (HR) and bacterial artificial chromosomes (BACs) have been traditionally used to manipulate the genome of herpesviruses and study their genes’ function [7]. Nowadays CRISPR/Cas9 is emerging as a new tool for DNA engineering in diverse organisms [8]. Large DNA viruses engineered by CRISPR/Cas9 have also been reported, including adenovirus, herpes simplex virus 1 (HSV-1) [9], and even PRV [10]. The RNA-guided Cas9 nuclease is used for efficient genome engineering in eukaryotic cells by simply specifying a 20-nt targeting sequence within its guide RNA (gRNA). An incision in the DNA mediated by Cas9/gRNA involves one of the two main repair mechanisms: HR or the error-prone non-homologous end-joining that frequently leads to small insertions or deletions [11]. Gibson assembly, developed by Dr. Daniel Gibson and colleagues, is a novel cloning method that eliminates the need for restriction enzyme digestion when cloning DNA fragments into a linearized vector [12]. It allows to assemble and clone oligos in a single step under isothermal conditions, and requires only a linearized vector and linear inserts with short sequences at each end that are homologous to one another.

The UL41 gene encodes the virion host shutoff (vhs) protein, a tegument protein in alphaherpesvirus virions [13]. This protein causes the dramatic degradation of both host and viral mRNA, as well as the shutoff of host cell protein synthesis in the early stage of infection [14, 15]. Furthermore, the endonuclease activity of vhs has been demonstrated using a rabbit reticulocyte in vitro translation system [16] and an in vitro luciferase reporter assay [17]. The roles of vhs in HSV-1 and HSV-2 have been studied extensively, however, relatively little is known about the vhs proteins of the other viruses. As for PRV vhs protein, it was also encoded by UL41 and shared approximately 38.4% identity at the amino acid level compared to that of HSV-1 UL41 [18]. Further research showed that purified vhs protein derived from European-American strain (TNL strain) that expressed in *E. coli* displays ribonuclease activity in vitro [19]. Subsequently Lin et al. generated a GFP-tagged UL41 null European-American strain of PRV and compared its vhs activity with that of wild type PRV (TNL strain), which demonstrated that PRV vhs is involved in host shutoff and also contributes to PRV virulence in a mouse model [20].

In this study, CRISPR/Cas9 technology was combined with the Gibson assembly method to enable manipulation and rescue of PRV mutants. Combined one-step Gibson assembly and incision with Cas9/gRNA largely enhanced the efficiency of constructing mutant viruses. Using this new approach, we were able to rapidly generate the UL41 null and revertant viruses of PRV variant. Compared to wild type, the UL41 null virus showed significantly smaller plaques and lower titers in vitro and impaired lethality and neuroinvasion in mice. Further the UL41 protein from different PRV strains exhibited unequal vhs activity in vitro, and which of JS-2012 showed significantly weaker vhs activity than that of European-American strains. In addition UL41 null virus can also significantly decrease the expression of host genes during the early period of infection, which suggests other viral factors may be also involved in host shutoff.

## Methods

### Cells and viruses

Porcine kidney (PK)-15, Madin-Darby bovine kidney (MDBK), African green monkey kidney (Vero) and human embryonic kidney 293 T (HEK293T) cells were cultured in Dulbecco’s modified Eagle’s medium (DMEM) supplemented with 10% fetal bovine serum. The cell cultures were maintained at 37 °C in a humidified 5% CO_2_ cell incubator. JS-2012 strain (GenBank Accession No. KP257591) was a PRV variant isolated in China and maintained in our laboratory [21], SC strain (GenBank Accession No. KT809429.1) is an earlier PRV isolate in China with its UL41 gene derived from Bartha vaccine strain (derivative of a virulent strain belonged to European-American strains) [22].

### Plasmids, oligos, and enzyme reagents

Plasmid pEGFP-C3 was purchased from Clontech (Shiga, Japan), pMD-18 T was purchased from Takara (Dalian, China). Plasmid pCMV-3 × Flag (Sigma-Aldrich, St. Louis, MO) was used for expression of UL41 protein of PRV JS-2012 and SC, by generating pCMV-3 × Flag-JS-2012 UL41 and pCMV-3 × Flag-SC UL41, respectively. The pLentiCRISPRv1 used in this study was a gift from Feng Zhang’s laboratory, and had been described previously [23]. All oligos or primers were synthesized by Genewiz (Suzhou, China). T4 polynucleotide kinase (PNK), *Eco*RV, *Bsm*BI, *Eco*RI, *Sal*I, Gibson Assembly® Master Mix, and T4 DNA ligase were purchased from New England Biolabs (Ipswich, MA).

### Donor plasmids construction

To insert the eGFP marker into the viral genome, we firstly generated a donor plasmid pBlue-eGFP-linker. Briefly, the eGFP cassette fragment (Additional file 1: Figure S1) and two fragments flanking the sequence of UL41 ORF (Additional file 1: Figure S1) were PCR amplified from pEGFP-C3 and the genome of JS-2012, respectively, using primer pairs EGFP-F&R, Up arm F&R, and Low arm F&R, respectively (Table 1). Gel purified PCR products were cloned into pMD-18 T and confirmed by DNA sequencing. Meanwhile, plasmid pBluescript II SK (+) was linearized by digestion with *Eco*RV. A total of 10 μL purified plasmid and PCR products mixture (plasmid:each product molar ratio = 1:5) was assembled in 10 μL Gibson Assembly® Master Mix and incubated at 50 °C for 1 h. A 10-μL aliquot of Gibson reaction mix was used to transform 100 μL DH5α competent *E. coli* cells following a standard transformation protocol. Positive clones were identified by targeted PCR of the eGFP sequence using primers GFP-F&R (Table 1), and were confirmed by DNA sequencing.

**Table 1:**
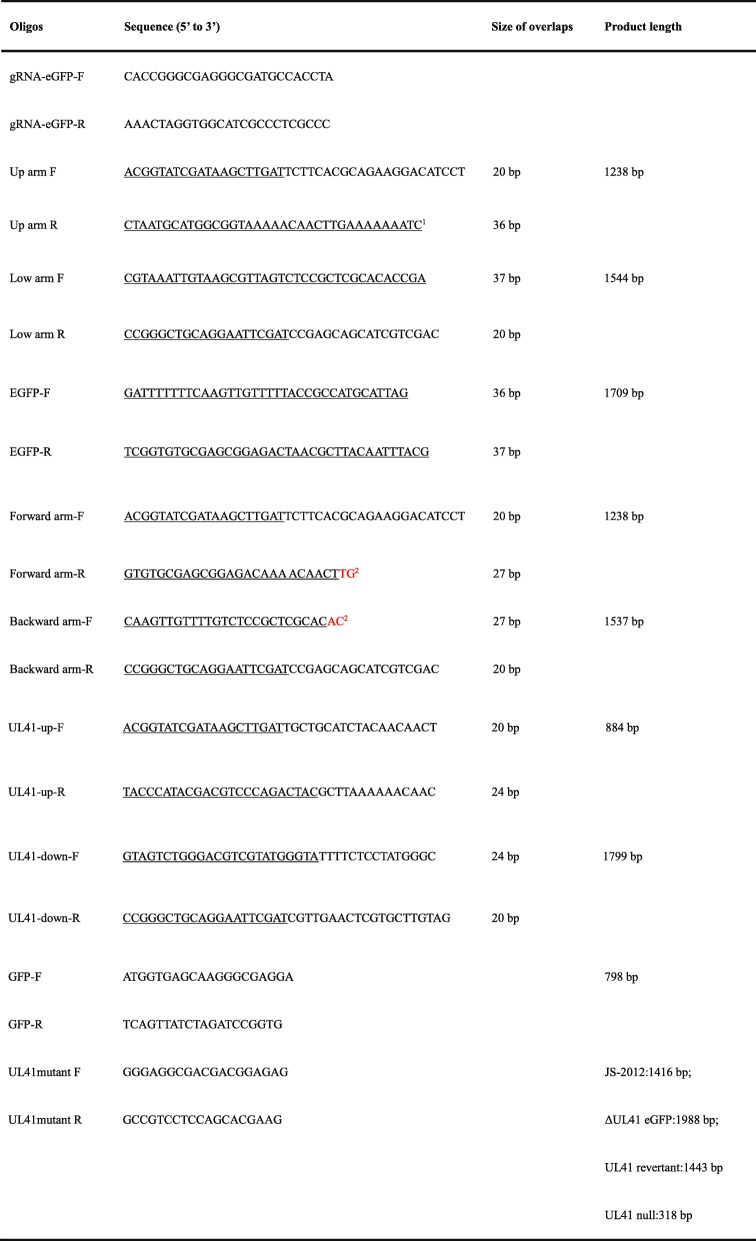
The oligos used for the generation and identification of mutant viruses

^a^Overlapping sequences between indicated adjacent DNA fragments are underlined

^b^Bases colored in red were included in overlaps but were excluded from primer oligos

To delete eGFP cassette from the virus via HR, another donor vector, pBlue-linker, was constructed. Two sequences (Additional file 1: Figure S1) flanking the UL41 ORF for HR were amplified from the genome of JS-2012 using primers Forward arm-F&R and Backward arm-F&R (Table 1). Similarly, to revert the UL41 on the viral genome, two PCR products (Additional file 1: Figure S1) containing the flanking region of UL41 and the UL41 ORF with an in-frame HA tag at its 3′ end were amplified by PCR from the genome of JS-2012 using primers UL41-up-F&R and UL41-down-F&R (Table 1). Then, the same assembly method described above was used to clone the related inserts into the linearized pBluescript II SK (+) vector. Positive clones were identified by PCR, and were confirmed by DNA sequencing.

### Construction of pLentiCRISPRv1-gRNA expressing vector

The specific gRNA for cleaving the coding sequence of eGFP was designed as previously described [24] and shown in Table 1. Briefly, oligos gRNA-eGFP-F and gRNA-eGFP-R were resuspended in RNase-free water to a final concentration of 100 μM respectively. Then, 1 μL sense oligo (gRNA-eGFP-F), 1 μL reverse oligo (gRNA-eGFP-R), 1 μL 10 × T4 PNK Reaction Buffer, 0.5 μL T4 PNK, and 6.5 μL RNase-free water were mixed. The resulting 10-μL reaction mixture was used for annealing and phosphorylation, and the double stranded gDNA thus obtained was cloned into *Bsm*BI-digested pLentiCRISPR vector by T4 DNA ligase. Correct insertion was further verified by DNA sequencing.

### Generation of virus mutants

**Fig. 1:**
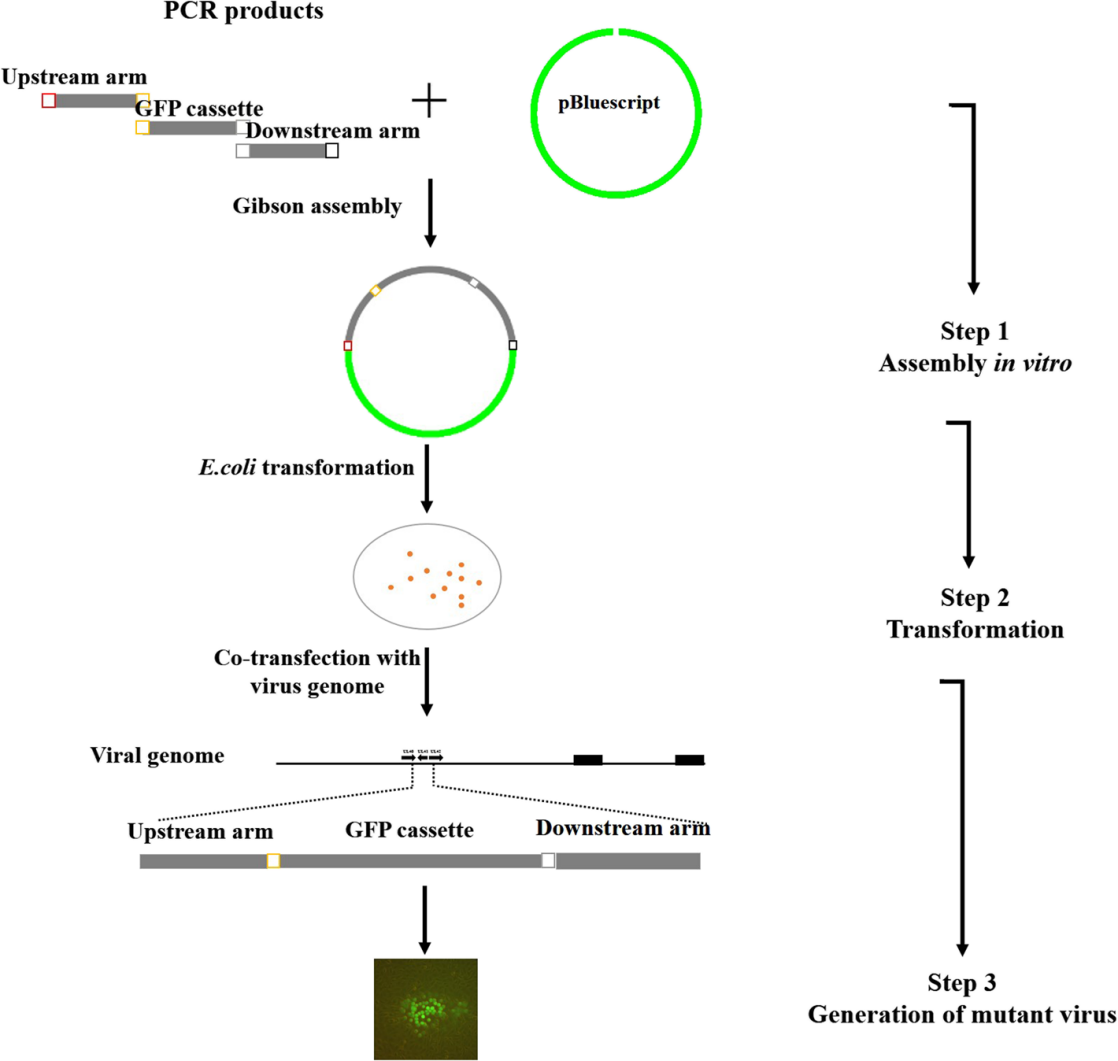
Schematic diagram of Gibson cloning combined with HR for generating the mutant virus

**Fig. 2:**
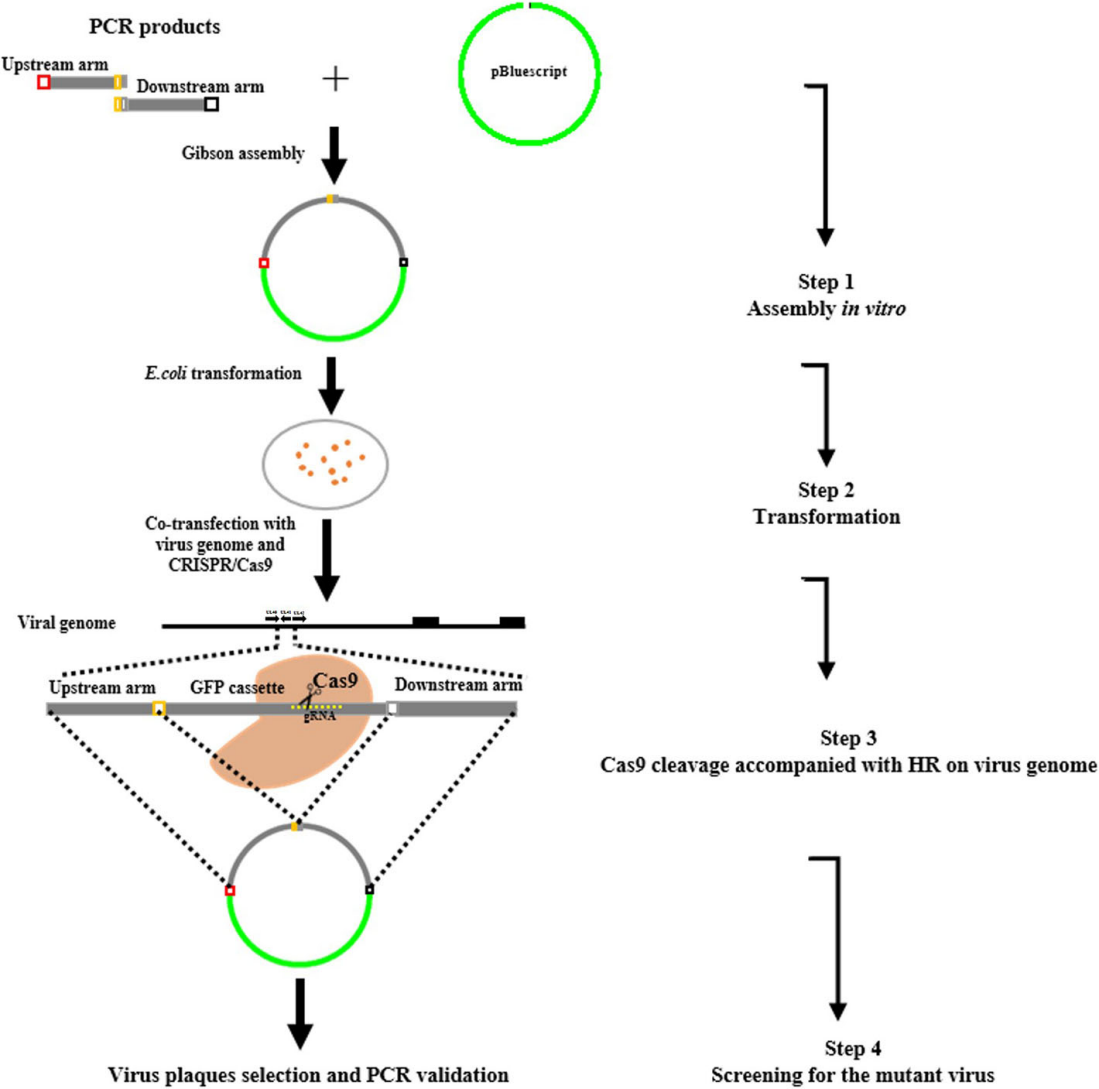
Gibson cloning and CRISPR/Cas9 combined with HR for generating the UL41 null and revertant viruses

To generate the UL41 null virus, we first had to introduce the eGFP cassette into the viral genome to replace the UL41 ORF by HR, and generate the ΔUL41 eGFP virus (Fig. 1). Then, the sequence flanking UL41 ORF and the sequence containing UL41 with an in-frame HA tag were cloned into pBluescript II SK (+) respectively, each of which was respectively co-transfected with the genome of ΔUL41 eGFP virus and pLentiCRISPRv1-gRNA-expressing vector targeting eGFP. Plaques exhibiting no fluorescent signal were picked under a fluorescence microscope, and were validated by PCR and sequencing to identify UL41 null virus and UL41 revertant virus (Fig. 2).

Briefly, PRV genomic DNA was extracted from infected Vero cells as described previously [25]. Then, the corresponding donor plasmid (2 μg) and viral genomic DNA from JS-2012, or genomic DNA from ΔUL41 eGFP virus (6 μg) and pLentiCRISPRv1-gRNA (1 μg) were co-transfected into Vero cells using FuGENE HD Transfection Reagent (Promega, Madison, WI). The transfected cells were harvested when approximately 80% cytopathic effect was observed. Recombinant viruses were selected through plaque purification and identified by the presence (ΔUL41 eGFP virus) or absence (UL41 null virus or UL41 revertant virus) of green fluorescence, specific PCR and Western-blot. Following three rounds of plaque purification, recombinant virus stocks were obtained.

### Animal experiments

To evaluate the pathogenicity of each virus in mice, six- to eight-week-old SPF BALB/c mice were divided into 7 groups (10 mice per group). In groups 1–3, mice were inoculated intranasally with 10 μL 10^5^ 50% tissue culture infective dose (TCID_50_) of PRV JS-2012, UL41 null virus and UL41 revertant virus under general aether anesthesia, respectively. In groups 4–6, mice were inoculated intranasally with 10 μL 10^4^ TCID_50_ of PRV JS-2012, UL41 null virus and UL41 revertant virus under anesthesia as above, respectively. Finally, mice in group 7 were inoculated with an equivalent volume of DMEM under anesthesia as above and constituted the control group. Animals were monitored and their health was recorded every day over a period of 10 days. Clinical signs and number of deaths were monitored daily after infection (Table 2).

**Table 2:** The infection progress of mice inoculated with JS-2012, UL41 null and revertant viruses

Viruses	Dose (TCID_50_)	Days post inoculation (dpi)									
		1	2	3	4	5	6	7	8	9	10
JS-2012	10^5^	–	+	+++ (−*)							
	10^4^	–	+	++	++ (−*)						
UL41 revertant	10^5^	–	+	+++ (−*)							
	10^4^	–	+	+	+++ (−*)						
UL41 null	10^5^	–	+	+++	+ (−*)						
	10^4^	–	+	+	+	+	–	–	–	–	–

“-”: No nervous signs such as unrest, tickle, depression, and biting the skin

“+”: Nervous signs such as unrest, tickle, depression, and biting the skin and < 50% mortality. “++”: Nervous signs such as unrest, tickle, depression, and biting the skin and 50% mortality. “+++”: Nervous signs such as unrest, tickle, depression, and biting the skin and > 50% mortality

“-*”: All mice died in this group

To analyze neuroinvasion of each virus in mice, mice were inoculated intranasally with 10 μL 10^4^ TCID_50_ of PRV JS-2012, UL41 null virus and UL41 revertant virus under anesthesia as above, and weight changes of infected mice were measured daily. Then survived mice were sacrificed at the indicated times. Mice trigeminal ganglia, brain stem, olfactory bulb, cerebrum and cerebel were harvested and frozen at 80 °C. Tissues were then thawed, homogenized, and frozen again. The homogenates were thawed, sonicated, centrifuged, and titrated by TCID_50_ assay on Vero cell monolayers.

All animal experiments were performed according to the Guide for the Care and Use of Laboratory Animals of the Shanghai Veterinary Research Institute, Chinese Academy of Agricultural Sciences, China.

### Functional assay for vhs activity

This assay was performed according to a previous study with a minor modification [17]. Briefly, the UL41 coding sequence of PRV JS-2012 was amplified by PCR using primers *Eco*RI-vhs-F (ggaattcaatggggctctttggcctttta) and *Sal*I-vhs-R (gcgtcgacttattttctcctatgggcgtt) and cloned into 3 × Flag-tagged expression vector pCMV-3 × Flag by *Eco*RI-*Sal*I digestion and T4 DNA ligation. The UL41 coding sequence of PRV SC was PCR-amplified by using primers SC-UL41-F (catcgatagatctgatagggctctttggcctttta) and SC-UL41-R (gagtcgactggtaccgatttattttctcctatgggcgt), and then cloned into linearized pCMV-3 × Flag by *Eco*RV cleavage in a Gibson assembly reaction. Afterwards the HEK293T cells were seeded into 6-well microtiter plates 24 h before transfection, then cells were transfected with 200 ng of SV40 luciferase reporter (pGL3-control) alone or in combination with pCMV-3 × Flag-JS-2012 UL41, pCMV-3 × Flag-SC UL41 or pCMV-3 × Flag empty vector at a dose of 2 μg using Lipofectamine™ 3000 reagent (Invitrogen, Carlsbad, CA). Thirty-six hours post transfection, cells were lysed with 200 μL of 1 × passive lysis buffer (Promega), and then luciferase readings were determined. Total protein concentration of the lysates was examined using the Bradford protein assay (Bio-Rad, Hercules, CA). Luciferase values from each sample were normalized to the respective total protein amounts. Expression of JS-2012 UL41-Flag or SC UL41-Flag was detected using a rabbit monoclonal anti-Flag antibody (Sigma-Aldrich); Expression of β-actin was detected using a monoclonal anti-β actin antibody (Sigma-Aldrich), and β-tubulin was used as a loading control.

### RNA extraction and RT-qPCR

Vero cells were mock-infected or infected with each PRV strain at a multiplicity of infection (MOI) of 10. On the other side, for cycloheximide (CHX) analysis Vero cells were untreated or treated with 100 μg/ml CHX prior to the infection as above. Then total RNA of each sample was extracted at 3 h post infection using the RNeasy Mini Kit (Qiagen, Hilden, Germany). RNA yield and quality were determined by UV spectroscopy.

Next, 1 μg of total RNA was DNase-treated using 2 U RNase-free DNase I (Ambion, Austin, TX). RNAs were then reverse-transcribed using the Superscript III RT-PCR System (Invitrogen, Carlsbad, CA) according to the manufacturer’s instructions for oligo(dT)-primed cDNA synthesis. cDNA was subject to qPCR using SYBR Premix Ex Taq (Takara) to measure the expression of host genes GAPDH and β-actin, or the viral genes UL41 and IE180; meanwhile the 28S rRNA was used as a reference gene in each run. The corresponding primers were shown in Table 3 and synthesized by Genewiz. The qPCR amplicons were detected using an Eppendorf (Hamburg, Germany) thermocycler in a final volume of 25 μL under the following cycle conditions: 95 °C for 2 min, 40 cycles of 95 °C for 15 s and 60 °C for 30 s. Relative quantities of each candidate gene were measured by the ΔΔCt method.

**Table 3:** Primer information for qRT-PCR amplification

Gene	Primer sequence (5′ to 3′)
28S rRNA	F: GGGCCGAAACGATCTCAACC
	R: GCCGGGCTTCTTACCCATT
GAPDH	F: ATCCTGGGCTACACTGAGGA
	R: TGTCGTACCAGGAAATGAGCT
β-actin	F: CTCTCTTCCAACCTTCCTTCC
	R: CAGACTCGTCATACTCCTGCTT
UL41	F: TGAAGAACGAGACGCGGG
	R: TGTGCTTCCAAAACAGGCCC
IE180	F: CATCGTGCTGGACACCATCGAG
	R: ACGTAGACGTGGTAGTCCCCCA

## Results

### Gibson assembly allows for efficient single-step cloning of multiple fragments into one vector

**Fig. 3:**
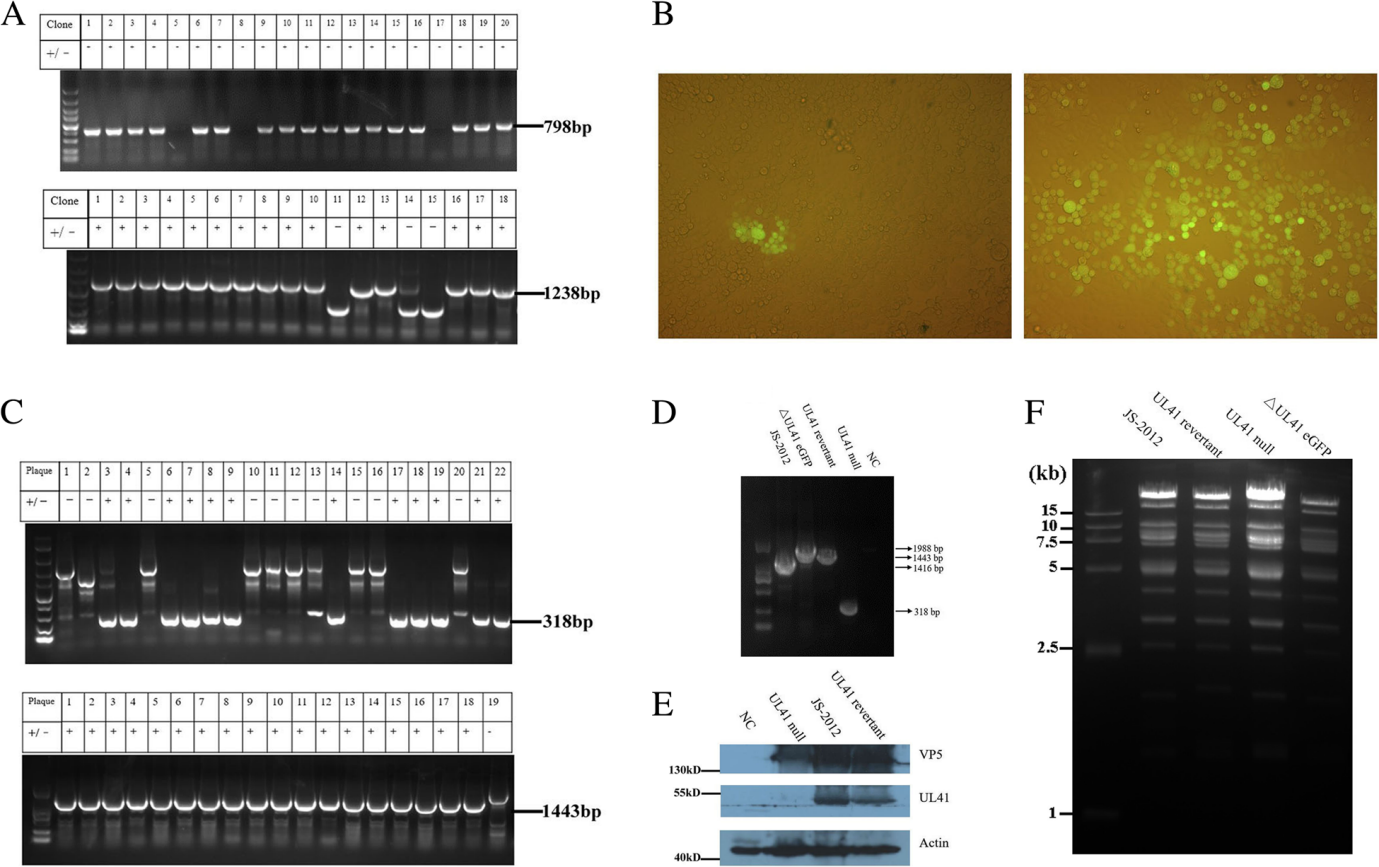
Generation and identification of UL41 mutant viruses. **a** Validation of Gibson-assembled donor plasmids pBlue-eGFP-linker (Top) and pBlue-linker (Bottom) by specific PCR. The sizes of positive PCR products were indicated on right side of each fig. **b** The donor plasmid pBlue-linker and genomic DNA of ΔUL41 eGFP virus were co-transfected into Vero cells together with or without pLentiCRISPRv1-gRNA. Then, cell culture supernatants were harvested when approximately 80% cytopathic effect was observed. Finally, eGFP protein expression was observed in Vero cells infected with virus supernatants collected from groups transfected with Cas9/gRNA (Left) or not (Right) (Magnification, 200×). **c** PCR validation of the generated UL41 null virus (Top) and UL41 revertant virus (Bottom). The sizes of positive bands were indicated on right side of each fig. **d** Identification of each mutant virus by specific PCR. Arrows on the right side indicated the expected sizes of PCR products. **e** The identification of UL41 protein expression during each virus infection by Western-blot. **f** *Bam*HI-based RFLP analysis of each PRV strain. The genomic DNA of each virus were prepared and digested respectively with *Bam*HI-HF (NEB) at 37 °C for 3 h. Digested mixtures were then resolved by 0.8% agarose-gel electrophoresis at 60 V for approximately 4 h, and visualized under ultraviolet light

To simplify the construction of donor plasmids, the Gibson assembly method was applied to clone multiple fragments into pBluescript in one step. The equal molar fragments (0.15 pmol each) and the linear vector (0.03 pmol) were mixed together for in vitro assembly and then subjected to transformation. Next, a number of *E. coli* colonies were randomly selected and analyzed by PCR. For the construction of pBlue-eGFP-linker, 17 out of 20 colonies were positive and confirmed by PCR using primers GFP-F&R; for the construction of pBlue-linker, 15 out of 18 colonies were proved positive by PCR using Forward arm-F&R (Fig. 3a); and for the donor plasmid used for UL41 reversion, almost all picked colonies (11/12) were positive (data not shown). DNA sequencing confirmed that each fragment was correctly and accurately inserted into the vector (data not shown). This provides strong evidence that Gibson assembly is a powerful tool for seamless single-step cloning of multiple fragments into the target vector.

### CRISPR/Cas9 incision enhances HR efficiency between donor plasmid and PRV genome

To enhance the probability of HR occurring between donor plasmid and PRV genome, CRISPR/Cas9 technology was adopted here to generate the UL41 null virus and UL41 revertant virus. Compared with the group without Cas9/gRNA, cleavage of the eGFP sequence by Cas9 inactivated eGFP expression in the majority of virus-infected cells in the group treated with Cas9/gRNA (Fig. 3b), indicating high incision efficiency of Cas9. For screening the UL41 null virus, a total of 22 viral plaques without green fluorescence were selected from the Cas9-treated group, and subjected to DNA extraction and PCR validation. Of these, 12 plaques were positive (54.5%) by PCR validation (Fig. 3c) and Sanger sequencing. And for the screening of UL41 revertant virus, 18 out of 19 viral plaques were positive by PCR validation (Fig. 3c). Then specific PCR (Fig. 3d) and Western-blot (Fig. 3e) both confirmed the null of UL41 or the reversion of UL41. Finally RFLP analysis indicated that all strains had the same RFLP pattern and maintained the integrity of each viral genome (Fig. 3f). Accordingly, CRISPR/Cas9 incision can largely enhance HR efficiency between donor plasmid and PRV genome.

### Characterization of UL41 mutants in PRV

**Fig. 4:**
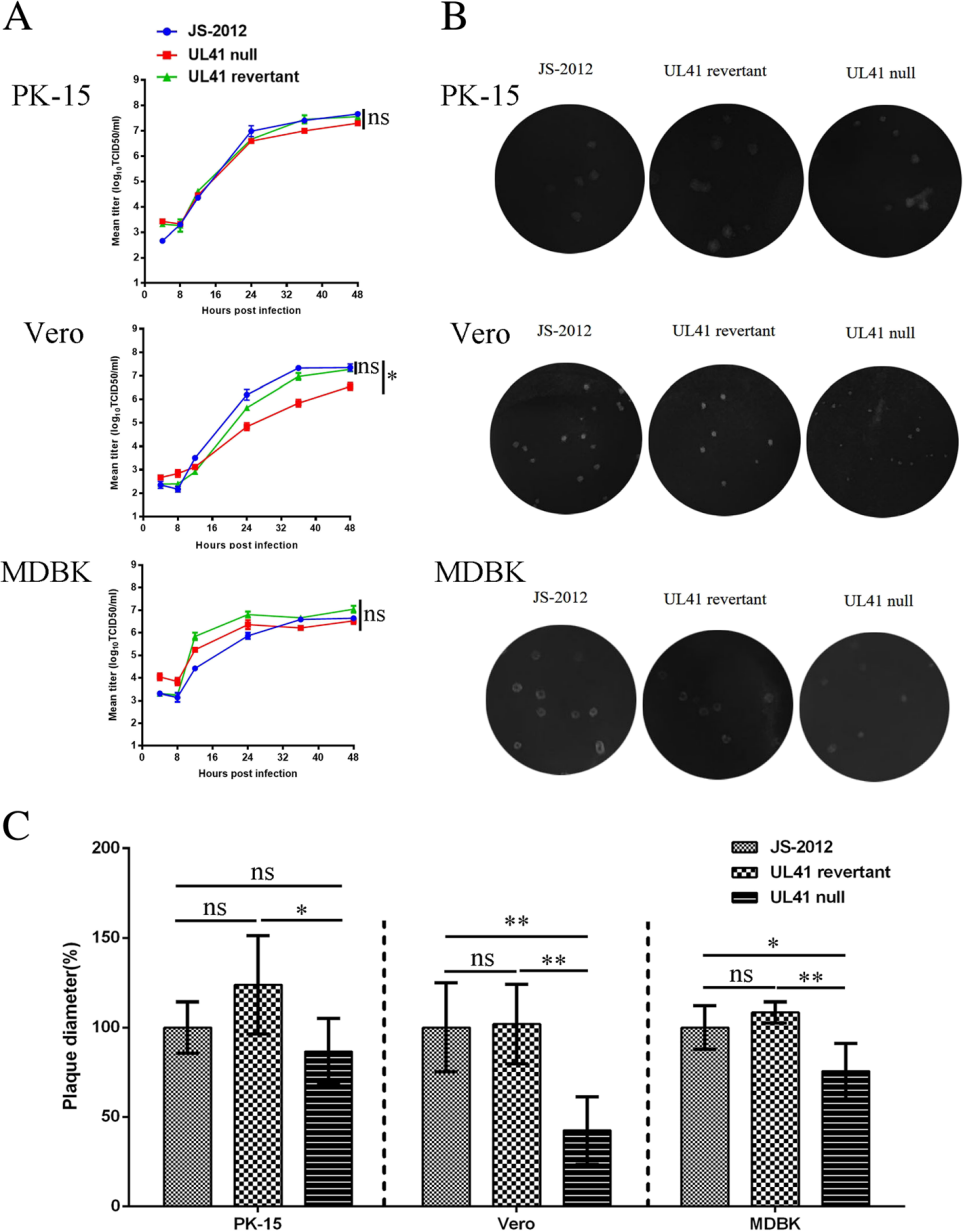
Characterization of UL41 mutant virus in vitro. **a** One-step growth curves. PK-15 (Top), Vero (Middle), and MDBK cells (Bottom) were infected with 1 MOI of each PRV strain. Cell culture supernatants were harvested at 4, 8, 12, 24, 36, and 48 h post infection. Virus titers were determined by TCID_50_ assay on Vero cells. The mean titers correspond to the averages of two independent experiments. And Two-way ANOVA was used for analyzing the data, *P < 0.05, ns was referred as no significance. **b** Plaque morphology of JS-2012, UL41 revertant virus and UL41 null virus in PK-15 (Top), Vero (Middle), and MDBK (Bottom) cells cultured at 37 °C for 4 days. **c** Relative plaque diameters of each virus were calculated and compared to those of PRV JS-2012. Meanwhile the average plaque diameter of PRV JS-2012 in each cells were set as 100%. And the Student’s *t*-test was used for analyzing the data, *P < 0.05, **P < 0.01, ns was referred as no significance

Previous studies on other herpesvirus UL41 genes, would suggest that the UL41 null PRV mutant would replicate less efficiently in vitro. As expected, the titer of UL41 null PRV was significantly lower than that of JS-2012 and the revertant virus in Vero cells (Fig. 4a), as indicated also by a significantly smaller plaque size (Fig. 4b, c). In particular, after 24 h post infection the titer of the UL41 null mutant was almost one order of magnitude lower than that of JS-2012 and revertant virus (Fig. 4a). While in PK-15 and MDBK cells the replication ability of UL41 null virus cells had not been greatly affected and showed only a little lower than that of JS-2012 and revertant virus (Fig. 4a). Plaque size of the UL41 null virus in MDBK cells was shown significantly smaller than that of JS-2012 and revertant virus, which of the UL41 null virus in PK-15 cells was comparable with that of JS-2012 but significantly smaller than that of revertant virus (Fig. 4c). By contrast, the replication and spread capacity of JS-2012 and its revertant virus was very similar in all the three cell lines (Fig. 4).

**Fig. 5:**
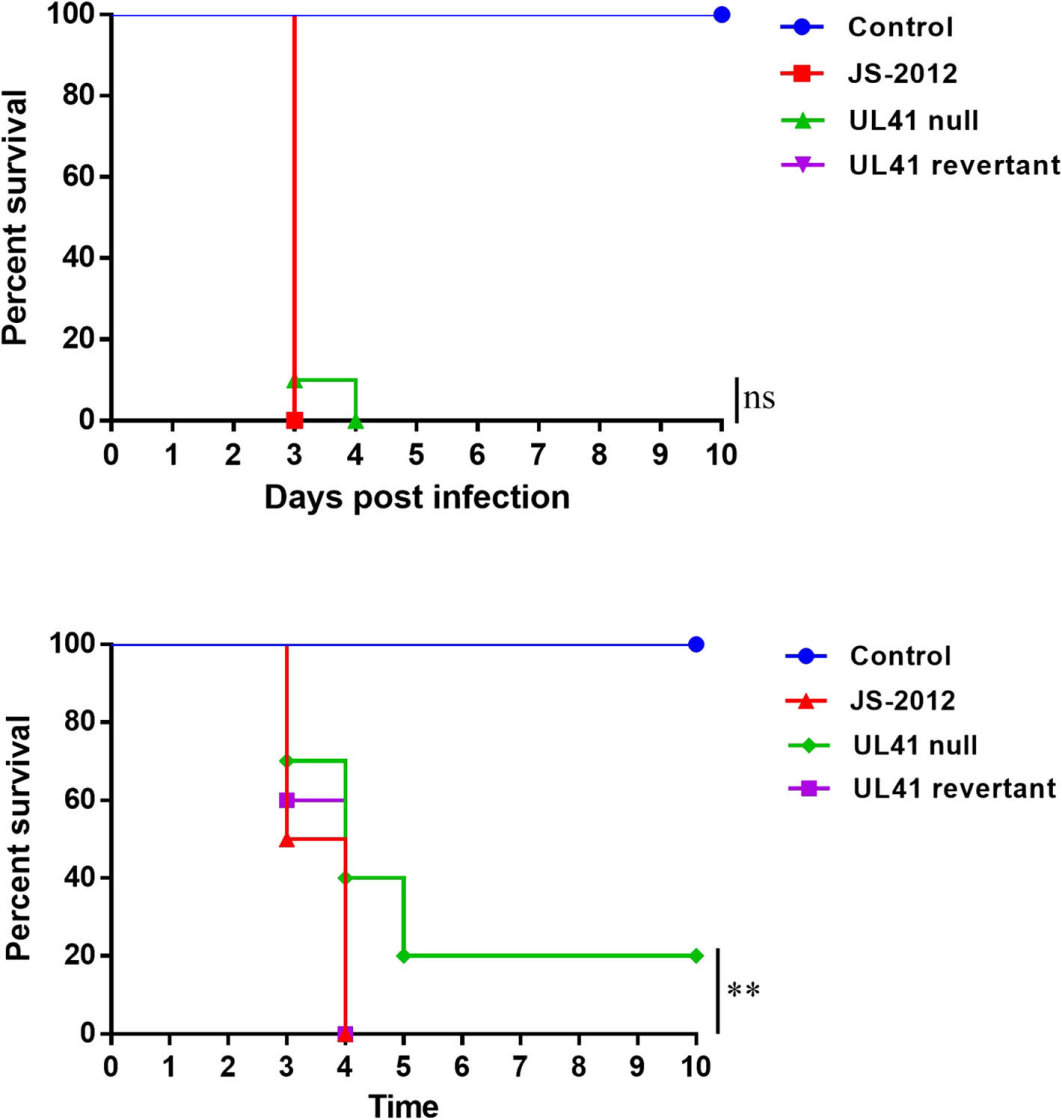
Survival percentages of mice. Mice were intranasally inoculated with 10^5^ TCID_50_ (Top) and 10^4^ TCID_50_ (Bottom) of JS-2012, UL41 revertant virus and UL41 null virus. Mice were observed every day and for a maximum of 10 days. The statistical significance between survival curves was evaluated by the log-rank (Mantel-Cox) test. *P* value < 0.05 was considered statistically significant, and *P < 0.05, **P < 0.01, ns was refered as no significance

To evaluate the role of UL41 in the pathogenicity of PRV variant in mice, BALB/c mice were inoculated intranasally with JS-2012, UL41 null virus and UL41 revertant virus. Mice inoculated with both 10^5^ TCID_50_ and 10^4^ TCID_50_ of the three viruses showed neurological symptoms, such as unrest, tickle, depression, and biting the skin (Table 2). At 10^5^ TCID_50_, mice all died by 3 days post inoculation (dpi) in the groups of JS-2012 and UL41 revertant virus but it was delayed by 1 day in the UL41 null virus group (Fig. 5). At 10^4^ TCID_50_, mice all died by 4 dpi in the groups of JS-2012 and UL41 revertant virus, while in the UL41 null virus group 2/10 mice survived (Fig. 5) and clinical symptoms delayed by 1 day than other groups (Table 2). Thus, UL41 deficiency could, to a certain extent, delay and reduce the clinical signs and lethality in mice.

### Neuroinvasion and kinetics of PRV JS-2012, UL41 revertant and null viruses

**Fig. 6:**
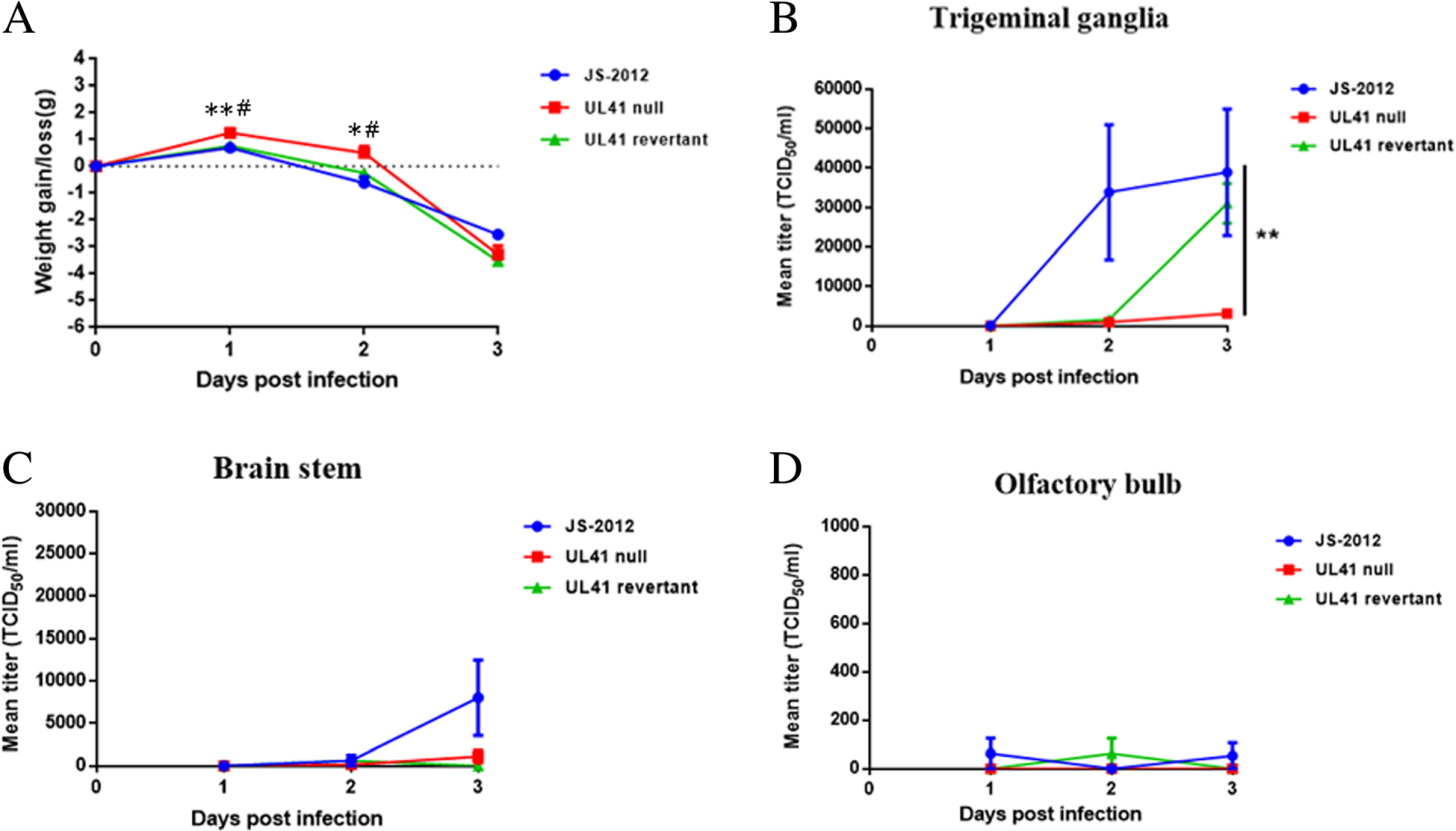
Acute replication in mice of PRV JS-2012, UL41 revertant virus and UL41 null virus. **a** Weight changes of infected mice during the period of acute infection. The data were presented as means ± SE for weight changes of 5 random selected mice per data point. Asterisk (*) indicated a significant difference between JS-2012 and UL41 null virus, and pound (#) indicated a significant difference between UL41 revertant and UL41 null viruses. Viral titers in the trigeminal ganglia (**b**), brain stem (**c**), Olfactory bulb (**d**) of mice infected with PRV strain JS-2012 (wild type), UL41 revertant virus or UL41 null virus were shown. The data represented means ± SE for 5 samples per data point. For panel **a**, the student’s *t*-test was used for analyzing the data; for panel **b**, two-way ANOVA was used for analyzing the data, **P < 0.01

To study the neuroinvasion of each PRV strains, viral replication in brain tissues and weight changes of mice infected with 10^4^ TCID_50_ of JS-2012, UL41 revertant and null viruses were assessed. After infection with PRV, the body weight of infected mice increased on 1 dpi while started to decrease on 2 dpi (Fig. 6a). But mice infected with UL41 null virus showed significantly reduced weight loss than those infected with JS-2012 or UL41 revertant virus (Fig. 6a). Further in virus-infected mice the titer of infectious virus could be detected in trigeminal ganglia (Fig. 6b), brain stem (Fig. 6c) and olfactory bulb (Fig. 6d), by contrast no infectious virus was detected in cerebrum and cerebel (data not shown). In trigeminal ganglia infectious virus of all three strains was detected, and on 3 dpi infectious virus was detected in 100% (5/5) of trigeminal ganglia of the three viruses (data not shown), and average JS-2012 titer was about 3.9 × 10^4^ TCID_50_/mL/tissue, average UL41 revertant virus titer was about 3.1 × 10^4^ TCID_50_/mL/tissue, while average UL41 null virus titer was significantly lower and was 3.2 × 10^3^ TCID_50_/mL/tissue (Fig. 6b). Compared to trigeminal ganglia, brain stem accommodated relatively lower titers of infectious virus and the infectious virus reached highest titers on 3 dpi (Fig. 6c), while infectious virus was only detected in 80% (4/5), 40% (2/5) and 0% (0/5) of brain stems of JS-2012, UL41 null virus and UL41 revertant virus respectively on 3 dpi (data not shown). In olfactory bulb the average titers of each virus at each timepoint were all lower than 10^2^TCID50/mL/tissue (Fig. 6d), and also infectious virus can not be detected in all mice in UL41 null virus group. In contrast no detectable infectious virus was detected in cerebrum and cerebel.

### Examination of host shutoff activity

**Fig. 7:**
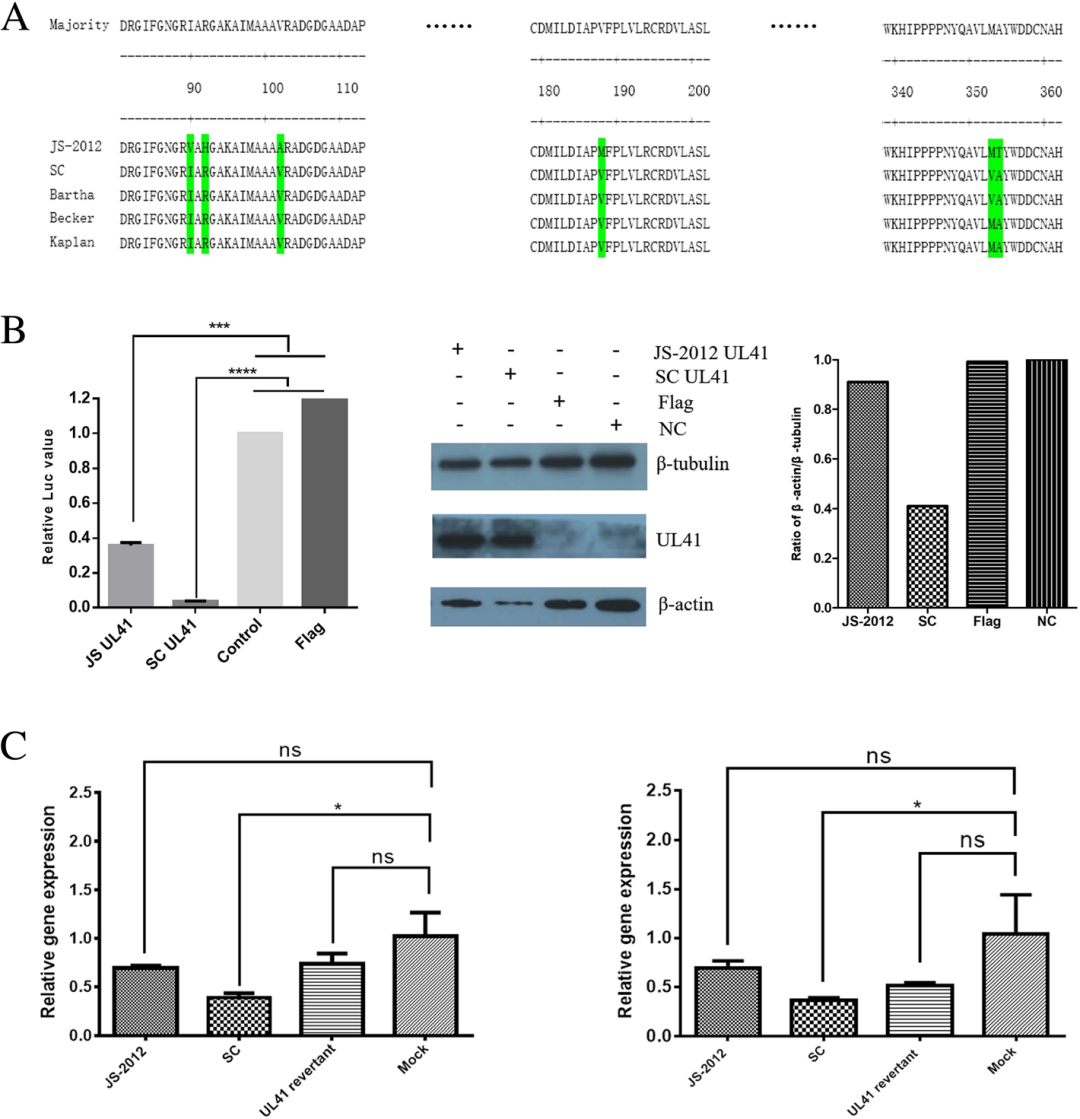
The functional analysis of host shutoff activity of UL41. **a** Multiple amino acid sequence alignment of the UL41 protein of different PRV strains. **b** Functional assay for vhs activity of the UL41 protein of different PRV strains. The HEK293T cells were co-transfected with 200 ng pGL3-control plasmid and 2 μg of plasmids expressing either empty vector (pCMV-3 × Flag), flag-tagged JS-2012 UL41, or flag-tagged SC UL41. After 36 h, the cells were harvested and luciferase readings were determined (Left). Data are representative of three independent experiments. Flag-tagged protein, β-actin and β-tubulin were detected by Western blot (Middle), and ratio of the expression of β-actin/β-tubulin was calculated by ImageJ software (Right). **c** The expression changes of β-actin (Left) and GAPDH (Right) at the 3 h post infection of 10 MOI of each virus infection or mock infection. All statistical analyses were performed using GraphPad Prism software with Student’s *t*-test, *P < 0.05, ns was refered as no significance

Multiple sequence alignment of PRV UL41 showed that the PRV variant JS-2012 exhibited several amino acids difference compared with European-American strains and the Chinese earlier strain PRV SC (Fig. 7a) who was a potential recombinant of Bartha vaccine and Chinese endemic strains [22]. To compare the vhs activity of UL41 protein between different PRV strains, the HEK293T cells were transfected with SV40 luciferase reporter plasmid in combination with either pCMV-3 × Flag-JS-2012 UL41 or pCMV-3 × Flag-SC UL41. Surprisingly, UL41 protein of PRV SC could significantly decrease the luciferase signal, which of PRV JS-2012 could not significantly reduce the expression of luciferase to the level as that of SC (Fig. 7b), suggesting that the vhs function of PRV JS-2012 UL41 might be deficient compared to other PRV strains. In addition, previous studies indicated PRV UL41 could reduce significantly the expression of β-actin [20]. Here the expression of β-actin in PRV UL41 transfection assay was also detected by Western blot, it showed SC UL41 could more significantly decrease the expression of β-actin than that of JS-2012 (Fig. 7b). Moreover, the changes in gene expression of β-actin and GAPDH in PRV-infected and mock-infected Vero cells were assessed. Similarly, the expression level of the two genes in SC-infected cells more significantly reduced than that in cells infected with JS-2012 and its revertant virus. But JS-2012 and its revertant virus could only slightly reduce the expression of host genes compared with mock-treated group (Fig. 7c).

**Fig. 8:**
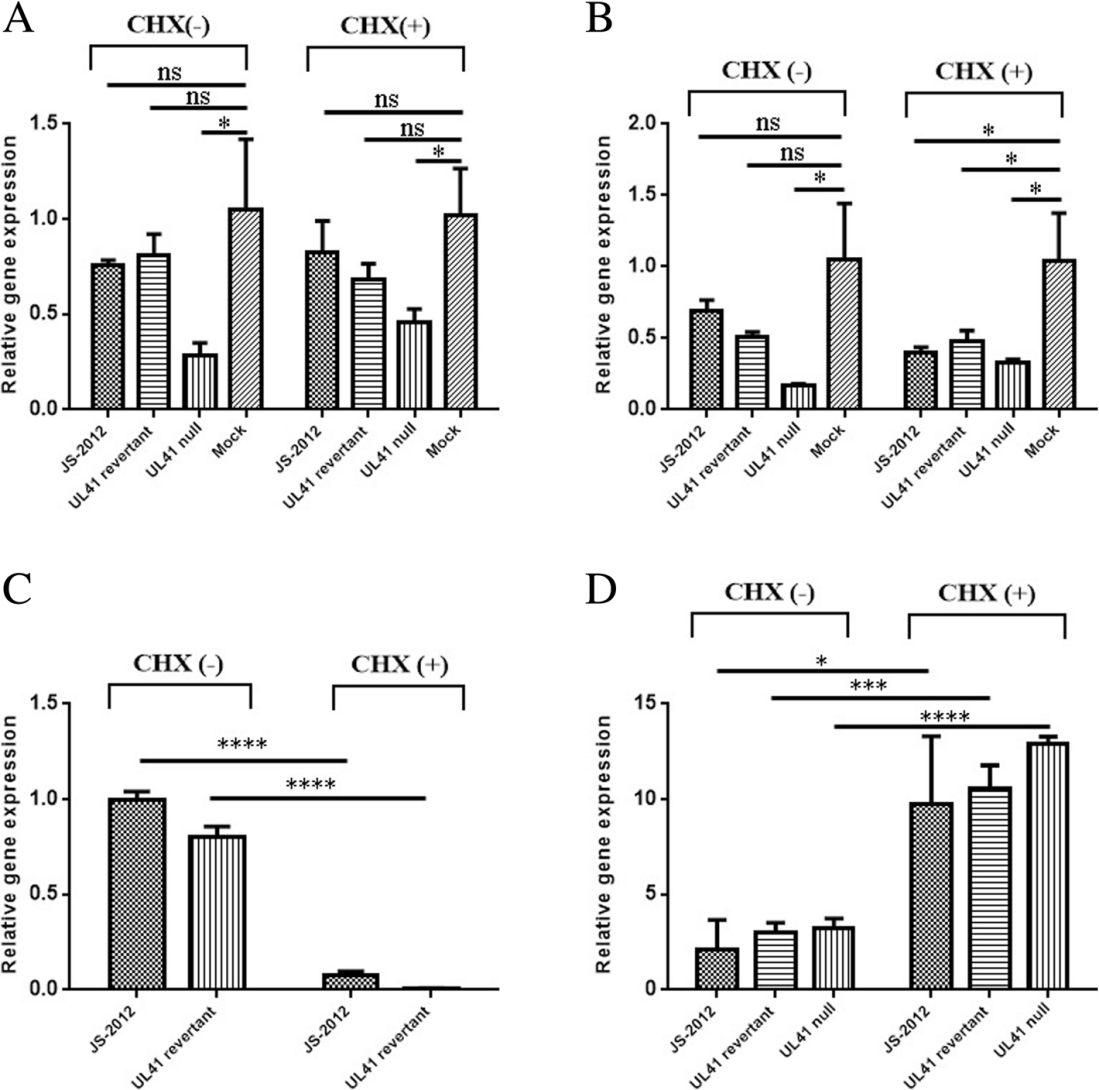
Expression changes of host and viral genes in Vero cells infected with 10 MOI each virus. At the timepoint of 3 h post infection, the expression levels of host genes β-actin (**a**) and GAPDH (**b**), and viral genes UL41 (**c**) and IE180 (**d**) were detected by RT-qPCR respectively. All statistical analyses were analysed with Student’s *t*-test, *P < 0.05, **P < 0.01, ***P < 0.001, ****P < 0.0001, ns was refered as no significance

In addition, surprisingly UL41 null virus infection could significantly downregulate the expression of β-actin (Fig. 8a) and GAPDH (Fig. 8b) at the timepoint of 3 h post infection, and this downregulation was more significant than that caused by JS-2012 and revertant virus; The CHX treatment on virus-infected cells could significantly reduce the viral UL41 gene expression by more than 10 folds (Fig. 8c), but has no significant effect on the downregulation of β-actin (Fig. 8a) and GAPDH (Fig. 8b) expression caused by virus infection. In contrast to the strong inhibition of UL41 transcription by CHX treatment, the only immediate-early gene (IE180) of each PRV strain was significantly upregulated by several folds than those without CHX treatment (Fig. 8d). Hence it seemed that UL41 protein of PRV variant had little to do with the host shutoff during the early period of virus infection.

## Discussion

Beginning in 2011, re-emerging PRV variants have been increasingly isolated in pig farms throughout China [4, 21, 26–28]. Genomic analyses have revealed that the variants are evolutionarily divergent from European–American strains [6]. Alphaherpesvirus UL41 was reported to encode a virion component that induced degradation of host mRNAs and shut off most host protein synthesis. In comparison, the function of the UL41 homolog of PRV has remained not so clear. Hence, in the present study, the UL41 null mutant of a PRV variant was generated by a novel method and was characterized both in vitro and in vivo.

Previously, the HR method dependent on the cell’s recombination and repair machinery was used to introduce a selectable marker into the viral genome, enabling enrichment and purification of the mutant virus. However, this process is laborious and time-consuming and the selectable marker carried by the mutant could affect the characteristics of the mutant virus. BAC technology is a very successful tool for studying herpesviruses because BACs could accommodate the large genomic DNA of these viruses [7]. Moreover, prokaryotic genome engineering can be adapted to alter BACs efficiently and in less time than recombination procedures in mammalian cells. However, construction and manipulation of viruses containing BACs are complex and might be difficult for inexperienced researchers.

Here, we adopted a novel approach to manipulate the PRV genome by combining CRISPR/Cas9 and Gibson assembly. Compared with traditional cloning methods such as those employing restriction enzymes, Gibson assembly could produce seamless, single-step, and efficient cloning of multiple fragments without introducing any additional sequence (Fig. 3a). In the present study, it only required a short overlapping sequence between adjacent DNA fragments, ranging from 20 to 37 bp (Table 1). Using Gibson assembly, we successfully cloned as many as three DNA fragments of different length (1238 bp, 1544 bp, and 1709 bp) into the linearized vector in one step. Through HR with the genome of JS-2012, the ΔUL41 eGFP virus was easily obtained by screening for viral plaques that exhibited green fluorescence. Subsequently, the same strategy was employed and successfully constructed two donor vectors for generation of UL41 null virus and UL41 revertant virus, by inserting the upstream (1238 bp) and downstream (1537 bp) regions of the UL41 ORF or two PCR products (884 bp and 1799 bp) containing UL41 ORF and HA-tag into pBluescript II SK (+) in a single step, respectively. It is hence proved that Gibson assembly based on homologous recombination can accomplish one-step insertion of viral DNA fragments into donor plasmids very efficiently.

Studies have revealed that CRISPR/Cas9-triggered site-specific cleavage could enhance HR efficiency in the genome of both prokaryotic and eukaryotic cells, and also DNA viruses [8, 29, 30]. Hence, we adopted the Cas9/gRNA technology for the generation of the UL41 null virus and UL41 revertant virus. To reduce the probability of off-target and non-target cleavage, a previously validated gRNA for cleaving the eGFP sequence was used (Table 1). As expected, incision of Cas9/gRNA in the eGFP cassette of the ΔUL41 eGFP virus significantly enhanced integration of donor plasmids into the virus genome via recombination repair, and further promoted the occurrence of HR in the generated virus stocks over 50% (Fig. 3c). In contrast, the occurrence of spontaneous HR in mammalian cells is often very low, ranging from 0.002 to 0.3% [31]. As in this study the merely HR efficiency was also very low (Fig. 3b), while the application of Cas9/gRNA could largely enhance the efficiency of HR and reduce the laborious process of selecting the recombinant virus (no fluorescent virus) from the fluorescent one (Fig. 3b, c).

The in vitro and in vivo properties of the UL41 null virus were further characterized. The UL41 gene was not essential for PRV growth in cell cultures, however its absence caused smaller plaques and lower titers in three different cell types. Particularly, in Vero cells the UL41 null virus presented more significantly impaired replication and spread capacity than that in the other two cells (Fig. 4), indicating that deletion of UL41 only impaired replication on cell lines of the specific species. And it can be speculated that the virus’s adaptability in different species is also different. As for in vivo characterization, the UL41 null virus seemed to impair lethality in mice, at least to a certain extent. It should be noted however, that the UL41 null strain still retained strong virulence in susceptible animals, with lethality in mice decreasing significantly only in the group challenged at a relatively lower dose (10^4^ TCID_50_). Compared to wild type PRV, the infection progress of mice inoculated with UL41 null virus was delayed by monitoring the clinical signs and number of deaths in mice, which suggested that the pathogenic capacity of UL41 null virus in mice was partially deficient. Further the neuroinvasive capacity of UL41 null virus, UL41 revertant virus and JS-2012 was assessed. During acute infection (from 1 dpi to 3 dpi) infectious virus can be detected in trigeminal ganglia, brain stem and olfactory bulb (Fig. 6). In trigeminal ganglia infectious virus of all three strains can be detected and exhibited very high titers, and on 3 dpi average titers of JS-2012 and UL41 revertant virus were significantly higher than that of UL41 null virus. By contrast, the PRV titers in brain stem and olfactory bulb were lower than that in trigeminal ganglia. Hence it can be seen that trigeminal ganglia was the main target of PRV infection, and UL41 null can significantly impair the replication and infection of PRV in trigeminal ganglia during acute infection.

The UL41 gene had long been known as the virion host shutoff gene in many alphaherpesviruses. Previous studies demonstrated that HSV-1 could induce the rapid degradation of pre-existing host mRNA including β-actin via the RNase activity of vhs protein [32–34]. To characterize the host shutoff activity of the UL41 gene of PRV variants, the luciferase assay was adopted here to detect the vhs activity of UL41 protein of PRV. Surprisingly, UL41 protein of PRV SC (an European-American-like strain) could significantly decrease the luciferase signal, whereas which of PRV JS-2012 could not significantly reduce the luciferase value to the level as that of SC (Fig. 7b), suggesting that the vhs function of PRV variant UL41 might be partially deficient compared to European-American strains. In addition, previous studies indicated PRV TNL (an European-American-like strain) infection could reduce significantly the expression of β-actin. Here the expression of β-actin in PRV UL41 transfection assay was also detected by Western blot, it showed SC UL41 could more significantly decrease the expression of β-actin than that of JS-2012 (Fig. 7b). Similarly, the mRNA expression level of β-actin and GAPDH in SC-infected cells more significantly reduced than that of JS-2012 and its revertant virus (Fig. 7c). But compared with mock-treated group, JS-2012 and its revertant virus could also slightly reduce the expression of host genes (Fig. 8). Therefore it can be seen that the UL41 protein from different PRV strains exhibited unequal vhs activity in vitro, and which of JS-2012 showed significantly weaker vhs activity than that of European-American strains. In addition, surprisingly either reduce UL41 protein expression by CHX or null UL41 on viral genome of PRV variant could not affect the downregulation of host genes’ expression during the early infection of each virus (Fig. 8), suggesting that UL41 protein of JS-2012 had no or very weak vhs activity in the early period of virus infection while other viral factors may be involved in host shutoff.

In conclusion, we combined CRISPR/Cas9 technology with the Gibson cloning method to generate the PRV UL41 null virus and UL41 revertant virus. Gibson assembly and incision of Cas9/gRNA largely enhanced the efficiency of constructing donor plasmids and homologous recombination, respectively. Compared to wild type (JS-2012), the generated UL41 null virus showed smaller plaques and lower titers in vitro*,* and impaired lethality in a mouse model. More importantly, null UL41 on viral genome of PRV variant could not affect the downregulation of host genes’ expression during early infection of the virus; Further, due to residue differences between UL41 proteins from genotype-specific strains, the UL41 protein from PRV variant showed significantly weaker vhs activity than that of PRV SC (European-American-like strain), which both suggested deficiency of vhs activity by the PRV variant UL41 protein.

## Conclusions

CRISPR/Cas9 combined with Gibson assembly efficiently generated UL41 null PRV. Compared to wild type, UL41 null PRV showed impaired both replication capability in vitro and lethality in vivo. Further UL41 protein of PRV variant showed significantly weaker vhs activity than that of PRV SC (European-American-like strain), suggesting the deficiency of vhs activity by the PRV variant UL41 protein.

## Additional file

Additional file 1: **Figure S1.** Sequence information about the fragments used to generate donor plasmids. (DOCX 19 kb)

## Abbreviations

BAC: Bacterial artificial chromosome
CHX: Cycloheximide
CRISPR: Clustered regularly interspaced short palindromic repeats
dpi: Days post inoculation
gRNA: Guide RNA
HR: Homologous recombination
HSV-1: Herpes simplex virus 1
HSV-2: Herpes simplex virus 2
MOI: Multiplicity of infection
PRV: Pseudorabies virus
qRT-PCR: Quantitative real-time reverse transcription-PCR
TCID_50_: 50% tissue culture infective dose
vhs: Virion host shutoff
