# Supplementary material for: Generation and characterization of UL41 null pseudorabies virus variant in vitro and in vivo
Source: Virol J. 2018 Aug 2;15:119. doi: 10.1186/s12985-018-1025-4 (PMC6090798; doi:10.1186/s12985-018-1025-4)
Supplement: Supplementary file 1 — Figure S1. Sequence information about the fragments used to generate donor plasmids. (DOCX 19 kb) [file 12985_2018_1025_MOESM1_ESM.docx]

**Additional files**

Additional file 1

**Figure S1.** Sequence information about the fragments used to generate donor plasmids.

**The sequence of eGFP cassette fragment amplified from plasmid pEGFP-C3**

gatttttttcaagttgtttttaccgccatgcattagttattaatagtaatcaattacggggtcattagttcatagcccatatatggagttccgcgttaca

taacttacggtaaatggcccgcctggctgaccgcccaacgacccccgcccattgacgtcaataatgacgtatgttcccat

agtaacgccaatagggactttccattgacgtcaatgggtggagtatttacggtaaactgcccacttggcagtacatcaag

tgtatcatatgccaagtacgccccctattgacgtcaatgacggtaaatggcccgcctggcattatgcccagtacatgacc

ttatgggactttcctacttggcagtacatctacgtattagtcatcgctattaccatggtgatgcggttttggcagtacat

caatgggcgtggatagcggtttgactcacggggatttccaagtctccaccccattgacgtcaatgggagtttgttttggc

accaaaatcaacgggactttccaaaatgtcgtaacaactccgccccattgacgcaaatgggcggtaggcgtgtacggtgg

gaggtctatataagcagagctggtttagtgaaccgtcagatccgctagcgctaccggtcgccaccatggtgagcaagggc

gaggagctgttcaccggggtggtgcccatcctggtcgagctggacggcgacgtaaacggccacaagttcagcgtgtccgg

cgagggcgagggcgatgccacctacggcaagctgaccctgaagttcatctgcaccaccggcaagctgcccgtgccctggc

ccaccctcgtgaccaccctgacctacggcgtgcagtgcttcagccgctaccccgaccacatgaagcagcacgacttcttc

aagtccgccatgcccgaaggctacgtccaggagcgcaccatcttcttcaaggacgacggcaactacaagacccgcgccga

ggtgaagttcgagggcgacaccctggtgaaccgcatcgagctgaagggcatcgacttcaaggaggacggcaacatcctgg

ggcacaagctggagtacaactacaacagccacaacgtctatatcatggccgacaagcagaagaacggcatcaaggtgaac

ttcaagatccgccacaacatcgaggacggcagcgtgcagctcgccgaccactaccagcagaacacccccatcggcgacgg

ccccgtgctgctgcccgacaaccactacctgagcacccagtccgccctgagcaaagaccccaacgagaagcgcgatcaca

tggtcctgctggagttcgtgaccgccgccgggatcactctcggcatggacgagctgtacaagtactcagatctcgagctc

aagcttcgaattctgcagtcgacggtaccgcgggcccgggatccaccggatctagataactgatcataatcagccatacc

acatttgtagaggttttacttgctttaaaaaacctcccacacctccccctgaacctgaaacataaaatgaatgcaattgt

tgttgttaacttgtttattgcagcttataatggttacaaataaagcaatagcatcacaaatttcacaaataaagcatttt

tttcactgcattctagttgtggtttgtccaaactcatcaatgtatcttaacgcgtaaattgtaagcgttagtctccgctcgcacaccga.

Note: The green highlighted sequence referred to eGFP ORF sequence, underlined sequences referred to sequences overlapped with the upstream and downstream sequences of UL41 ORF.

**The upstream sequence of PRV UL41 ORF for generation of pBlue-eGFP-linker**

acggtatcgataagcttgattcttcacgcagaaggacatcctgcactactacatcgagc aggagtgcatcgaggtggtgcactcgcgcgtgtacagcgccatccagctga tgctgttccgcggcgacgccgcagcgcgcgagcgctacgtgcgcgcggcgctgcgggacgaggccatccg

ccgcaaggtggagtggctcgactcgcgcgtcgccgagtgcgcctccgtcgcggaaaagtacctgctcatgatcctcatcg

agggcatcttcttcgcctcctcgttcgcctccatctcgtacctccgcacgcacaacctgtttgtggtggcgtgccagtcc

aacgacttcatcagccgcgacgaggccatccacacctcggcctcgtgctgcatctacaacaactacctgggggacgcccc

gcgccccgacgaggcccgcatccaccagctcttcgccgaggcggtggagatcgagtgcgagtttctgcgggcgcgcgccc

cgcgcgacagcctcctgctggacctgccggccatcatctcgtacgtgcgctacagcgcggaccggctgctgcaggccatc

ggcgcgagcccgctctttggcgcgcccgcccccgcggcggactttcccatggcgctgatggtcgccgagaagcacaccaa

ctttttcgagcggcgcagcaccaactacacggggaccgtcgtgaacgacctgtagcgcccagcggcggcgacccccgctc

tcccccctcctcctctctctgcgacaaaacactaataaagcgttgagacactagcgcgcgcctccagccgtcatccttgg

ggcgagggtgggacgggaacggggatgggagagtggggtggggagagtgcggggatgatgacgggggaagggagatgggg

ctgacgggggtggactgggactggggaaggatgatggcggccggcgcagagtgggaggcggtgaggtggacggcgggtgg

gacggcggggggaccgatgtggggcggcagaggggcggcagaggggcggcagaggggcggcagaggggcggcagaggggc

ggcagaggggcggctgaggggcggcagaggggcggcggcgaggtgagacggagcccgcggacggagtggccggcgggggg

aggcgacgacggagagagaaggtgggggagagggcgagcatcacacgggccgcacaaccgaggatttttttcaagttgtt

tttaccgccatgcattag.

Note: The starting and ending site of this sequence in viral genome was 49,508-50,709 bp. The underlined sequences referred to sequences overlapped with pBluescriptⅡSK(+) and eGFP cassette fragment respectively.

**The downstream sequence of PRV UL41 ORF for generation of pBlue-eGFP-linker**

cgtaaattgtaagcgttagtctccgctcgcacaccgaggcgccggtctaaaatacg catgtggcggggcggggccacccgcgcatataagccgggcggtgattggtctg gcgcacaccgcccgcccgagcccgctcgcccgccgcgatgtcgctgttcgacgacggcctcgaggacct

ggaccgccaccccacccacgcgcaccacccggcgcaggtgatccacgacgggcccttcgtgctggaggacggcgagcccc

tgcagcgcaccggcatgctggtgctcagcgacgagcacctggagcacgcgcgcgccgccatcgccccgctcgccgcgcac

ctcgcgcacgccttcctcgtcttcagcgaggccgggctgctggtgcacgccagcgtgcgcggcgagcaggtctacgtgac

cctggccccggaccagttcagcacgttcgtgtggagcgggccccaggccgtgttcctgggcaacgtcgacggcagcggcg

gcgtgctcgacgcgctcaaggtcgaccggcggcggaccgtcttcaacgtcaccttcgaggtgtacggcgccttcccggcg

cggctgctgacgcggcgcgcgtactttgcggacgcgggcctcctcgcggcggggcccggctccccgagcgtcgcctgcgt

ctacaagcacgagttcaacgactactgcatcatgctcccctcgcgggcgcccgacgtgagcctgacgctctcgcgccccc

aggtggccaagctcgccgccgtggcgaagggcgccgcggccgggacgaccttcgcgctcgcccgcggcctcgacttctcc

gtctcctccagcgccggggtcgtgaccttcccggcgcgcgaccacgacgggaccgccgtgctggagcgcgccagccggcg

gcgccagggcgtcgacgcggtcggcgcgacggagcccttcgccatgacgctcgaggcggcgcacgggctgctgacgctgc

tgcagcggctgcgggccgggaacgccgagctcacgttcaactttttcacgacgccgcggcaggcgcccctgttcagcgtg

accacctgcggcccggtgcgggcgaccaccttcttcttctgcgcgcccgccgaccccgccaccgtgcccgccgcccccga

gggcgccgccgccaccgtcgccgccgcctgcggggcgggcgcgtccgccgcctcccccgccgcgggggacaagcggcccg

ccgccccgcgcatgtacacgcccatcgccaagcgcccgcggaccgcctcgggggaagggggccacgcctacggagattta

ttctaataaagtgagcaggtgtataaaagagaggtcgcacctccgcgtctttactcgccgtcgcgatgatctcccggggg

aacggagggggttgccgccccggcgagccatgctggcgctgcgcgctcgagtccacccgctgcatcacgctgatgggcgt

gctcgtcgcgctcctcgccgcctgcatgctgtccgtcccgcccgcggcgtcgacgatgctgctcggatcgaattcctgcagcccgg.

Note: The starting and ending site of this sequence in viral genome was 51,808-53313 bp. The underlined sequences referred to sequences overlapped with eGFP cassette fragment and pBluescriptⅡSK(+) respectively.

**The upstream sequence of PRV UL41 ORF for generation of pBlue-linker**

acggtatcgataagcttgattcttcacgcagaaggacatcctgcactactacatcgagcaggagtgcatcgagg

Tggtgcactcgcgcgtgtacagcgccatccagctgatgctgttccgcggcgacgccgc agcgcgcgagcgctacgtgcgcgcggcgctgcgggacgaggccatccg

ccgcaaggtggagtggctcgactcgcgcgtcgccgagtgcgcctccgtcgcggaaaagtacctgctcatgatcctcatcg

agggcatcttcttcgcctcctcgttcgcctccatctcgtacctccgcacgcacaacctgtttgtggtggcgtgccagtcc

aacgacttcatcagccgcgacgaggccatccacacctcggcctcgtgctgcatctacaacaactacctgggggacgcccc

gcgccccgacgaggcccgcatccaccagctcttcgccgaggcggtggagatcgagtgcgagtttctgcgggcgcgcgccc

cgcgcgacagcctcctgctggacctgccggccatcatctcgtacgtgcgctacagcgcggaccggctgctgcaggccatc

ggcgcgagcccgctctttggcgcgcccgcccccgcggcggactttcccatggcgctgatggtcgccgagaagcacaccaa

ctttttcgagcggcgcagcaccaactacacggggaccgtcgtgaacgacctgtagcgcccagcggcggcgacccccgctc

tcccccctcctcctctctctgcgacaaaacactaataaagcgttgagacactagcgcgcgcctccagccgtcatccttgg

ggcgagggtgggacgggaacggggatgggagagtggggtggggagagtgcggggatgatgacgggggaagggagatgggg

ctgacgggggtggactgggactggggaaggatgatggcggccggcgcagagtgggaggcggtgaggtggacggcgggtgg

gacggcggggggaccgatgtggggcggcagaggggcggcagaggggcggcagaggggcggcagaggggcggcagaggggc

ggcagaggggcggctgaggggcggcagaggggcggcggcgaggtgagacggagcccgcggacggagtggccggcgggggg

aggcgacgacggagagagaaggtgggggagagggcgagcatcacacgggccgcacaaccgaggatttttttcaagttgtt

ttgtctccgctcgcacac.

Note: The starting and ending site of this sequence in viral genome was 49,508-50,709 bp. The underlined sequences referred to sequences overlapped with pBluescriptⅡSK(+) and downstream fragment of UL41 ORF respectively.

**The downstream sequence of PRV UL41 ORF for generation of pBlue-linker**

caagttgttttgtctccgctcgcacaccgaggcgccggtctaaaatacgcatgtggcggggcggggccacccgcgcatataagccgggcgg

tgattggtctggcgcacaccgcccgcccgagcccgctcgcccgccgcgatgtcgctgttcgacgacggcctcgaggacct

ggaccgccaccccacccacgcgcaccacccggcgcaggtgatccacgacgggcccttcgtgctggaggacggcgagcccc

tgcagcgcaccggcatgctggtgctcagcgacgagcacctggagcacgcgcgcgccgccatcgccccgctcgccgcgcac

ctcgcgcacgccttcctcgtcttcagcgaggccgggctgctggtgcacgccagcgtgcgcggcgagcaggtctacgtgac

cctggccccggaccagttcagcacgttcgtgtggagcgggccccaggccgtgttcctgggcaacgtcgacggcagcggcg

gcgtgctcgacgcgctcaaggtcgaccggcggcggaccgtcttcaacgtcaccttcgaggtgtacggcgccttcccggcg

cggctgctgacgcggcgcgcgtactttgcggacgcgggcctcctcgcggcggggcccggctccccgagcgtcgcctgcgt

ctacaagcacgagttcaacgactactgcatcatgctcccctcgcgggcgcccgacgtgagcctgacgctctcgcgccccc

aggtggccaagctcgccgccgtggcgaagggcgccgcggccgggacgaccttcgcgctcgcccgcggcctcgacttctcc

gtctcctccagcgccggggtcgtgaccttcccggcgcgcgaccacgacgggaccgccgtgctggagcgcgccagccggcg

gcgccagggcgtcgacgcggtcggcgcgacggagcccttcgccatgacgctcgaggcggcgcacgggctgctgacgctgc

tgcagcggctgcgggccgggaacgccgagctcacgttcaactttttcacgacgccgcggcaggcgcccctgttcagcgtg

accacctgcggcccggtgcgggcgaccaccttcttcttctgcgcgcccgccgaccccgccaccgtgcccgccgcccccga

gggcgccgccgccaccgtcgccgccgcctgcggggcgggcgcgtccgccgcctcccccgccgcgggggacaagcggcccg

ccgccccgcgcatgtacacgcccatcgccaagcgcccgcggaccgcctcgggggaagggggccacgcctacggagattta

ttctaataaagtgagcaggtgtataaaagagaggtcgcacctccgcgtctttactcgccgtcgcgatgatctcccggggg

aacggagggggttgccgccccggcgagccatgctggcgctgcgcgctcgagtccacccgctgcatcacgctgatgggcgt

gctcgtcgcgctcctcgccgcctgcatgctgtccgtcccgcccgcggcgtcgacgatgctgctcggatcgaattcctgcagcccgg.

Note: the starting and ending site of this sequence in viral genome was 51,808-53313 bp. The underlined sequences referred to sequences overlapped with upstream fragment of UL41 ORF and pBluescriptⅡSK(+) respectively.

**The two PCR products for generation of donor plasmid for UL41 reversion**

**Sequence 1:**

acggtatcgataagcttgattgctgcatctacaacaactacctgggggacgccccgcgccccgacgaggcccgcatccaccagctcttcgccgaggcggtggagatcgagtgcgagtttctgcgggcgcgcgccccgcgcgacagcctcctgctggacctgccggccatcatctcgtacg

tgcgctacagcgcggaccggctgctgcaggccatcggcgcgagcccgctctttggcgcgcccgcccccgcggcggacttt

cccatggcgctgatggtcgccgagaagcacaccaactttttcgagcggcgcagcaccaactacacggggaccgtcgtgaa

cgacctgtagcgcccagcggcggcgacccccgctctcccccctcctcctctctctgcgacaaaacactaataaagcgttg

agacactagcgcgcgcctccagccgtcatccttggggcgagggtgggacgggaacggggatgggagagtggggtggggag

agtgcggggatgatgacgggggaagggagatggggctgacgggggtggactgggactggggaaggatgatggcggccggc

gcagagtgggaggcggtgaggtggacggcgggtgggacggcggggggaccgatgtggggcggcagaggggcggcagaggg

gcggcagaggggcggcagaggggcggcagaggggcggcagaggggcggctgaggggcggcagaggggcggcggcgaggtg

agacggagcccgcggacggagtggccggcggggggaggcgacgacggagagagaaggtgggggagagggcgagcatcaca

cgggccgcacaaccgaggatttttttcaagttgttttttaagcgtagtctgggacgtcgtatgggta.

**Sequence 2:**

gtagtctgggacgtcgtatgggtattttctcctatgggcgttacagtcgtcccagtacgtcatgaggacggcctggtagttcggaggcggaggtatgtgcttccaaaacaggcccgccagctccttcgcccgcgtctcgttcttcacgtgccgcagcagcgagccaaagaccttgttcacgtcc

gagggctcctgcacgatggggacgcgccgcagcacgctcaggtgcccgtgccgcgccggcgtgagcatggccaccacgtg

ctggatgaactcgcgctcggcgcggcgcgagcgcgcgtccccggccgccggcagcagcgcgagcgccttgccggcgtcgc

gcatgatgtcggggcagcgggacgcgtacttgagcttgacgtcctcgggcgccacctcgcggccctccagcgactcggcc

acctgctgcaccgagtccacgtccggcgcccggtgcaggtccgtgtggcagcgcacgaacgcggccaggaactccgagta

gtccacccccaggctcgcgaggacgtcgcggcagcgcagcaccagcgggaacatgggcgcgatgtcgaggatcatgtcgc

acccggtgaggatcatgtccgtgtcggtcgtgtgcacctgcgcgaccgtgttggtgtggtagaggttggcgcagacgtcg

tccgcctccatgtccgacacgtccacgtaggcgtagcccatgtggcggatgaggttgacgcagaggcggtggacgatgcg

cggcgcgtgcagcatcgtgctccagcgcggccgcggcggcgcgtcggcggccccgtcgccgtcggcgcgcgccgccgcgg

ccatgatggccttggcgccgtgcgcgacgcggccgttcccgaagatgccgcggtccgagacgaagatggggaagtaggtg

cgcttgtgcagcatgcgcagcaggcgcagcaggcagcgggcggtcgtggtcgcgttgtcctcggtcgtctcctggtagtg

cttctccatgagggtgtacatgacgttccacaggtcgatggcgatgggcgtgaggaccccgggcggcgtggagatggcct

ccgagcggaccagacgatggcgatgtgcgtactttaaaaggccaaagagccccatgtctccgctcgcacaccgaggcgcc

ggtctaaaatacgcatgtggcggggcggggccacccgcgcatataagccgggcggtgattggtctggcgcacaccgcccg

cccgagcccgctcgcccgccgcgatgtcgctgttcgacgacggcctcgaggacctggaccgccaccccacccacgcgcac

cacccggcgcaggtgatccacgacgggcccttcgtgctggaggacggcgagcccctgcagcgcaccggcatgctggtgct

cagcgacgagcacctggagcacgcgcgcgccgccatcgccccgctcgccgcgcacctcgcgcacgccttcctcgtcttca

gcgaggccgggctgctggtgcacgccagcgtgcgcggcgagcaggtctacgtgaccctggccccggaccagttcagcacg

ttcgtgtggagcgggccccaggccgtgttcctgggcaacgtcgacggcagcggcggcgtgctcgacgcgctcaaggtcga

ccggcggcggaccgtcttcaacgtcaccttcgaggtgtacggcgccttcccggcgcggctgctgacgcggcgcgcgtactttgcggacgcgggcctcctcgcggcggggcccggctccccgagcgtcgcctgcgtctacaagcacgagttcaacgatcgaattcctgcagcccgg

Note: the starting and ending site of the two sequences in viral genome was 49,873-52,467 bp. The underlined sequences referred to overlapped sequences with each adjacent fragment. The green highlighted sequence referred to HA-tag sequence.
